# Factors associated with participant retention in a clinical, intensive, behavioral weight management program

**DOI:** 10.1186/s40608-015-0041-9

**Published:** 2015-03-01

**Authors:** Amy E Rothberg, Laura N McEwen, Andrew T Kraftson, Nevin Ajluni, Christine E Fowler, Nicole M Miller, Katherine R Zurales, William H Herman

**Affiliations:** Department of Internal Medicine, University of Michigan, 24 Frank Lloyd Wright Drive, Ann Arbor, MI 48106 USA; Department of Epidemiology, University of Michigan, 1000 Wall Street, Ann Arbor, MI 48105 USA

**Keywords:** Obesity, Weight loss, Weight loss maintenance, Very low calorie diet, Financial incentives, Depression

## Abstract

**Background:**

We sought to identify factors associated with participant retention in a 2-year, physician-lead, multidisciplinary, clinical weight management program that employs meal replacements to produce weight loss and intensive behavioral interventions and financial incentives for weight loss maintenance. We studied 270 participants enrolled in 2010 and 2011. Sociodemographic factors, health insurance, distance traveled, body mass index, comorbidities, health-related quality-of-life, and depression were explored as potential predictors of retention.

**Results:**

Mean age was 49 ± 8 years and BMI was 41 ± 5 kg/m^2^. Retention was excellent at 3 months (90%) and 6 months (83%). Attrition was greatest after participants were transitioned to regular foodstuffs and fell to 67% at 12 months and 51% at 2 years. Weight decreased by 15 ± 12 kg and BMI decreased by 5.1 ± 4.0 kg/m^2^ in 2-year completers. Older age, lower baseline BMI, and financial incentives for program participation were independently associated with retention. Fewer depressive symptoms at baseline were associated with retention.

**Conclusions:**

This multidisciplinary, clinical, weight management program demonstrated high retention and excellent outcomes. Older age at baseline, less extreme obesity, and financial incentives were associated with program retention.

## Background

Retention is a major challenge for behavioral weight management programs and directly impacts their effectiveness. In a systematic review of major commercial weight loss programs, Tsai and Wadden concluded that behavioral programs that employ low-calorie exchange diets and weekly group sessions can produce 5% weight loss at 2 years [1]. More recently, Spring and colleagues described patient retention in a clinical weight management program implemented in the Veterans Health Administration that focuses on diet, physical activity, and behavior modification [2]. Sixty-four percent of participants remained in the program after the first session, 36% remained in the program at ~3 months, and only 26% remained in the program at ~6 months [2]. In multivariate analyses, older veterans, women veterans, and obese and morbidly obese veterans compared to overweight veterans were more likely to remain in the program. Use of incentives or rewards was also associated with greater retention [2]. More intensive weight management programs led by physicians and other health care providers that employ weekly classes, group sessions, and very low-calorie diets (VLCD) using meal replacement products have been shown to produce 15% to 25% weight loss at 3 to 6 months but are associated with higher attrition and a high probability of ≥50% weight regain at 1 to 2 years, especially when implemented in real world clinical settings and not as a part of a randomized controlled clinical trial [1].

The purpose of this study was to describe factors associated with retention in a 2-year, physician-led, multidisciplinary, intensive behavioral weight management program that employed VLCD using meal replacement products to induce weight loss, and intensive behavioral interventions and financial incentives to facilitate long-term weight loss maintenance. This program was a self-sustaining clinical program implemented without the supplemental resources often available in clinical trials. We hypothesized that by identifying patient-level factors and clinical outcomes associated with program retention, we could modify the program to improve future effectiveness and efficiency.

## Methods

The University of Michigan Weight Management Program (WMP) is a two-year clinical program that employs intensive energy restriction for the first 3 to 6 months to promote 15% weight loss, followed by behavioral change and physical activity counseling to promote longer-term weight loss maintenance. Prior to enrollment, patients are asked to review an online orientation session that discusses the scope of the obesity problem, the biology of obesity, and the rationale, approach, and requirements of the program. Patients who elect to enroll must sign a contract agreeing to attend ≥80% of scheduled appointments. Those who fail to comply are terminated from the program. Patients are seen by a physician for an initial assessment, at one month, and quarterly thereafter. Patients are seen and weighed weekly by a dietitian during the first month, and monthly thereafter. The entire 2-year program involves 11 visits with a physician and 26 visits with a dietitian. Laboratory monitoring is performed at baseline prior to dietary intervention, at one month during VLCD in patients at high risk for metabolic derangements (e.g. chronic kidney disease, history of heart failure), and every 3 to 6 months thereafter depending on the participant’s comorbid health conditions.

Over the first 3 to 6 months of the program, participants consume a very low calorie diet (VLCD, 800 kcal/day) in the form of total meal replacement and are asked to gradually increase low to moderate intensity physical activity to 40 minutes per day. They are also asked to keep diaries to record the number of meal replacement shakes and soups they consume, deviations from the prescribed diet, feelings of hunger, satiety, and mood, and physical activity. These diaries are reviewed each week with the dietitian either in person, by telephone, or by email. Following the initial 3 to 6 months, participants are transitioned to regular food stuffs. An exercise physiologist is available to provide a one-time, one-hour, exercise consultation to develop an activity program that incorporates patient preferences. Participants are subsequently asked to perform 40 to 90 minutes of moderate to vigorous physical activity at least 4 days per week.

In this report, we analyzed data from participants who enrolled in the WMP between January 1, 2010 and December 31, 2011. All program participants had BMI ≥32 kg/m^2^ with one or more comorbidities or BMI ≥35 kg/m^2^. The majority (96%) of participants were insured by Healthy Blue Living (HBL), a commercial health insurance plan that includes strong financial incentives for patients, providers, and employers to work towards improved health in six high-impact areas including obesity [3]. The remainder had commercial health insurance plans that covered the WMP but not copayments for dietitian visits. All participants paid for meal replacements which averaged $300/month. HBL members who adhered to program requirements received enhanced benefits: co-payments were waived for dietitian visits and were reduced for prescription drugs. All participants who did not attend ≥80% of scheduled appointments were terminated for non-adherence. HBL members who were terminated reverted to standard health insurance benefits. The difference in out-of-pockets costs between HBL enhanced and standard benefits is approximately $800 per participant per year.

We assessed health-related quality-of-life using the EQ-5D, a 5-item generic health utility instrument in which a summary score of 1.0 represents the best imaginable health state and a score of 0 represents a health state equivalent to death [4]. We also assessed health-related quality-of-life with the Impact of Weight on Quality-of-Life-Lite (IWQOL-lite), a 31-item, self-reported measure of obesity-specific quality-of-life that is responsive to weight loss and weigh gain [5]. For participants enrolled after May 2010, we also assessed depressive symptom severity with the Inventory of Depressive Symptomatology-Self Report (IDS-SR), a 30-item instrument that provides a total score ranging from 0 to 84 [6]. A score of 0 to 13 corresponds to no depressive symptoms and scores of 14 to 25, 26 to 38, 39 to 48, and 49 to 84 correspond to mild, moderate, severe, and very severe depressive symptoms.

The demographic and clinical characteristics of the study population were described using means ± standard deviation (SD) or number and percentage (%). Participants who did not have any visits on or after 3, 6, 12, 18, and 24 months were defined as having withdrawn at each time point. Participants were divided into groups according to the length of time they were enrolled in the weight management program. Short-term completers were defined as those who made at least one visit on or after 6 months, and 2 year completers were defined as those who made at least one visit 22 to 24 months after enrollment. The demographic and clinical characteristics of the study population stratified by short-term and 2-year completion were compared using t-tests for continuous variables and chi-square tests for categorical variables. A multivariate logistic model was constructed to predict 2 year completion using variables that were significant (p ≤0.1) in bivariate analyses (with the exception of IDS-SR because of missing values). The model included age in years, race (nonHispanic white vs. other), insurance type (Healthy Blue Living vs. other), BMI (kg/m^2^), EQ-5D score, EQ-5D anxiety or depression score (moderate or severe vs. non), and IWQOL-lite score. In the model, age in years, BMI (kg/m2), EQ-5D score, and IWQOL-lite score were defined as continuous variables.

Analyses were performed using SAS version 9.3 (SAS Institute, Cary, NC). The study was reviewed and approved by the University of Michigan Institutional Review Board and all participants provided written informed consent.

## Results

Between January 1, 2010 and December 31, 2011, 300 participants enrolled in the University of Michigan Weight Management Program (WMP). Thirty participants involuntarily withdrew before 2-years of follow-up: 25 because of changes in health insurance, 3 because they moved out of the area, 1 because of pregnancy, and 1 because of death. These participants were not considered in this report. Of the 270 enrollees who could have potentially completed the program, 137 (51%) completed it and 133 (49%) failed to complete it. Of those who failed to complete the program, 67 (50%) were terminated for non-adherence, 40 (30%) stopped attending despite repeated attempts to contact them, and 26 (20%) withdrew for other reasons including 3 who elected to undergo bariatric surgery.

Table 1 shows the baseline characteristics of the 270 program participants. Mean age was 49 ± 8 years, 48% were men, and 85% were non-Hispanic whites. In general, the population was married, highly educated, and employed. Most (96%) had Healthy Blue Living (HBL) insurance, which provided a financial incentive for program participation. The mean distance from home to the intervention site was 35 ± 18 kilometers. Mean BMI was 41 ± 5 kg/m^2^ at baseline and comorbidities were common. At baseline, 29% had diabetes, 53% had hypertension, 50% had dyslipidemia, 34% had obstructive sleep apnea, 30% had osteoarthritis, and 21% had depression. One-quarter to nearly two-thirds of participants reported at least some problems with mobility, usual activities, pain, and anxiety or depression. Health-related quality-of-life as assessed by the EQ-5D was generally good although depressive symptoms as assessed by both the EQ-5D and the Inventory of Depressive Symptomatology-Self Report (IDS-SR) were common as was the negative impact of weight on health-related quality-of-life as assessed by the IWQOL-lite.

**Table 1:** Baseline characteristics of program participants*

	**Total N = 270**
**Age (years)**	49 ± 8
**Sex**	
Male	130 (48%)
Female	140 (52%)
**Race/ethnicity**	
NonHispanic White	229 (85%)
Other	41 (15%)
**Marital status (missing = 2)**	
Married or life partner	237 (88%)
Other	31 (12%)
**Education (missing = 2)**	
High school or less	9 (3%)
Some college	48 (18%)
College graduate	116 (43%)
Professional degree	95 (35%)
**Employment (missing = 5)**	
Employed	214 (81%)
Retired/keeping house/other	51 (19%)
**Insurance group**	
Healthy Blue Living (HBL)	259 (96%)
Other	11 (4%)
**Distance from home to clinic (km) (missing = 2)**	35 ± 18
**Body mass index (kg/m** ^**2**^ **)**	41 ± 5
**Comorbidities (missing = 2)**	
Diabetes	78 (29%)
Hypertension	142 (53%)
Dyslipidemia	136 (50%)
Obstructive sleep apnea	93 (34%)
Osteoarthritis	80 (30%)
Depression	57 (21%)
**EQ-5D (missing = 3)**	0.84 ± 0.13
Mobility problems	68 (25%)
Self-care problems	7 (3%)
Usual activities problems	64 (24%)
Pain problems	174 (65%)
Anxiety/depression problems	86 (32%)
IWQOL-lite** (missing = 3)	68 ± 18
IDS-SR*** (missing = 65)	14 ± 9

*Results are expressed as mean ± standard deviation or number (%).

**Impact of Weight on Quality-of-Life-Lite.

***Inventory of Depressive Symptomatology-Self Report.

Table 2 shows participant retention at 3, 6, 12, 18, and 24 months. In general, retention was excellent at 3 months (90%) and 6 months (83%). Attrition was greatest between 6 and 12 months, after the transition from VLCD to regular food stuffs. Retention was 67% at 12 months, 55% at 18 months, and 51% at 2 years.

**Table 2:** **Participant retention over time (n = 270)**

	**Retained**	Withdrew*
**At 3 months**	244 (90%)	26 (10%)
**At 6 months**	223 (83%)	47 (17%)
**At 12 months**	181 (67%)	89 (33%)
**At 18 months**	149 (55%)	121 (45%)
**At 24 months**	137 (51%)	133 (49%)
**Mean number of program visits attended**	21 ± 2	10 ± 5

*Withdrawal defined as no visits on or after the month specified.

Table 3 shows the baseline characteristics of participants who completed 6 or more months in the program (n = 223) compared to those who were terminated or withdrew before 6 months (n = 47), and those who completed 2 years in the program (n = 137) compared to those who were terminated or withdrew before 2 years (n = 133). Participants who completed 6 months and 2 years in the program were significantly older and had lower baseline BMIs compared to non-completers at 6 months and 2 years. Compared to 6 month non-completers, participants who completed 6 months in the program were more likely to be highly educated and employed (p < 0.1). Participants who completed 2 years in the program had fewer baseline problems with anxiety and depression as assessed by the EQ-5D, and exhibited less impact of both depressive symptoms and weight on health-related quality-of-life (p < 0.05). Compared to 2-year non-completers, participants who completed 2 years in the program were more likely to be insured by HBL which provided financial incentives for ongoing program participation, to be non-Hispanic White, and to have better baseline health-related quality-of-life as assessed by the EQ-5D (p < 0.1). Sex, marital status, distance from home to the intervention site, and baseline comorbidities were not associated with retention at either 6 months or 2-years.

**Table 3:** **Baseline characteristics of completers and noncompleters**

	Short-term completer* N = 223	**Short-term noncompleter N = 47**	**p-value**	**2-year completer N = 137**	**2-year noncompleter N = 133**	**p-value**
**Age (years)**	50 ± 8	46 ± 9	0.0033	51 ± 8	47 ± 8	0.0002
**Race/ethnicity**			0.6995			0.0490
NonHispanic White	190 (85%)	39 (83%)		122 (89%)	107 (80%)	
Other	33 (15%)	8 (17%)		15 (11%)	26 (20%)	
**Education (missing = 2)**			0.0644			0.6864
High school or less	8 (4%)	1 (2%)		4 (3%)	5 (4%)	
Some college	34 (15%)	14 (30%)		24 (18%)	24 (18%)	
College graduate	102 (46%)	14 (30%)		64 (47%)	52 (40%)	
Professional degree	78 (35%)	17 (37%)		45 (33%)	50 (38%)	
**Employment (missing = 5)**			0.0880			0.4907
Employed	181 (83%)	33 (72%)		106 (79%)	108 (83%)	
Retired/keeping house/other	38 (17%)	13 (28%)		28 (21%)	23 (18%)	
**Insurance group**			0.4122			0.1119
Healthy Blue Living	215 (96%)	44 (94%)		134 (98%)	125 (94%)	
Other	8 (4%)	3 (6%)		3 (2%)	8 (6%)	
**Body Mass Index (kg/m** ^**2**^ **)**	40 ± 5	43 ± 7	0.0177	40 ± 5	42 ± 6	0.0015
**EQ-5D (missing = 3)**	0.84 ± 0.13	0.82 ± 0.14	0.3662	0.85 ± 0.12	0.82 ± 0.14	0.0745
Mobility limitations	58 (26%)	10 (21%)	0.4675	36 (27%)	32 (24%)	0.6494
Self-care limitations	7 (3%)	0	0.6103	4 (3%)	3 (2%)	0.7242
Usual activities	52 (24%)	12 (26%)	0.7823	32 (24%)	32 (24%)	0.9179
Pain	141 (64%)	33 (70%)	0.4239	87 (64%)	87 (66%)	0.8017
Anxiety/depression	69 (31%)	17 (36%)	0.5221	36 (27%)	50 (38%)	0.0500
IWQOL-lite** (missing = 3)	68 ± 17	64 ± 19	0.1441	70 ± 17	65 ± 18	0.0138
IDS-SR*** (missing = 65)	14 ± 9	17 ± 10	0.0826	13 ± 9	16 ± 10	0.0331

*Short-term completers defined as those with a visit at or after 6 months.

**Impact of Weight on Quality-of-Life-Lite.

***Inventory of Depressive Symptomatology-Self Report.

When factors associated with completing the 2-year program (p ≤ 0.1) were entered into a multivariable logistic regression model to predict retention, only three factors were independently and significantly associated with retention. These were older age, lower baseline BMI, and having health insurance that provided financial incentives for program participation (Table 4). Race, baseline health-related quality-of-life, and symptoms of anxiety or depression as assessed by the EQ-5D were not independently associated with completing the 2-year program. In sensitivity analyses, we included all variables in Table 3 in three separate models, each including only one HRQOL variable (EQ-5D score, moderate or extreme anxiety/depression as assessed by the EQ-5D, or IWQOL-lite score). The results were not different.

**Table 4:** **Factors independently associated with program retention at 2 years**

**Factor**	**Wald chi-square**	**p-value**	**Odds ratio**	**95% wald confidence limits**
**Intercept**	0.22	0.6358	--	--
**Older age (years)**	10.31	0.0013	1.06	1.02-1.09
**Other race**	1.31	0.2516	0.65	0.31-1.36
**Healthy Blue Living Insurance**	4.51	0.0337	5.92	1.16-22.68
**Higher BMI (kg/m** ^**2**^ **)**	6.05	0.0139	0.93	0.87-0.99
**Higher EQ-5D score**	0.24	0.6221	1.87	0.16-22.68
**Moderate or severe Anxiety/Depression (EQ-5D)**	0.89	0.3459	0.72	0.37-1.42
Higher IWQOL-lite*	0.04	0.8514	1.00	0.98-1.02

*Impact of Weight on Quality-of-Life-Lite.

**Figure 1:**
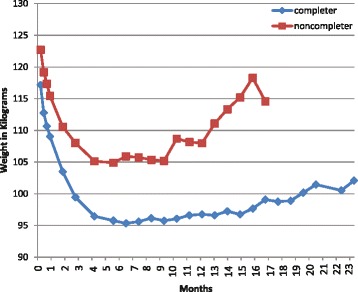
**Change in weight (kg) over time for 2-year program completers and noncompleters.**

Figure 1 shows change in weight in kilograms over time for program completers and non-completers. The percent of baseline body weight lost at 6 months was significantly associated with program retention at 6 months (16.0 ± 7.3% vs. 9.3 ± 4.6%, p < 0.0049). The percent of baseline body weight lost at 6 months was also associated with program retention at 2 years (17.7 ± 6.4% vs. 12.9 ± 7.8%, p < 0.0001).

Table 5 shows the changes in outcomes among participants who completed the 2-year program. Weight decreased by 15 ± 12 kg and BMI decreased by 5.1 ± 4.0 kg/m^2^. There were significant improvements in both systolic blood pressure and HDL-cholesterol despite less frequent use of antihypertensive and lipid lowering medications. Mean EQ-5D score improved by 0.06 ± 0.12. A change greater than 0.03 is considered to be clinically meaningful. Nineteen percent of participants who reported at least some limitations in mobility, 2% who reported some limitations in self-care, 22% who reported some limitations in usual activity, 33% who reported some pain, and 13% who reported some anxiety or depression at baseline reported no limitations at follow-up 2-years later. Mean IWQOL-lite scores increased by 15 ± 13: a 7.7 to 12 point increase in the total score is considered to be a clinically meaningful improvement. The impact of depressive symptoms also decreased significantly between baseline and follow-up and the mean number of antidepressant medications prescribed increased significantly. Among 2-year program completers, 56% of those with diabetes reported improvements at follow-up as did 23% of those with hypertension, 13% of those with dyslipidemia, 27% of those with obstructive sleep apnea, 4% of those with osteoarthritis, and 52% of those with anxiety or depression (as measured by the EQ-5D subscore).

**Table 5:** **Changes in outcomes among participants who completed 2 years in the program (n = 137 unless otherwise specified)**

	**Change from baseline**	**p-value**
**Weight (kg)**	−15 ± 12	<0.0001
**Body mass index (kg/m** ^**2**^ **)**	−5.1 ± 4.0	<0.0001
**Systolic blood pressure (mmHg)**	−5 ± 16	0.0002
**HDL cholesterol (mg/dl) (n = 119)**	11 ± 9	<0.0001
**Mean number of medications**		
Blood pressure	−0.34	<0.0001
Lipid lowering	−0.09	0.0068
Depression	0.09	0.0011
**EQ-5D (n = 87)**	0.06 ± 0.12	<0.0001
IWQOL-lite* (n = 87)	15 ± 13	<0.0001
IDS-SR** (n = 70)	−2.6 ± 7.0	0.0026

*Impact of Weight on Quality-of-Life-Lite.

**Inventory of Depressive Symptomatology-Self Report.

## Discussion

In this study, we demonstrated both high retention and excellent weight loss outcomes among moderately and severely obese patients, many of whom met BMI criteria that would potentially qualify them for bariatric surgery. Instead, they enrolled in a 2-year, physician-led, multidisciplinary, weight management program that employed meal replacement products to induce weight loss and intensive behavioral interventions and financial incentives to promote long-term weight loss maintenance. Retention was 83% at 6 months and 51% at 2 years. The greatest attrition occurred after the transition from VLCD to regular food stuffs. Our results are encouraging in that an analysis of data from the National Weight Control Registry found that individuals who succeed in maintaining their weight loss for 2 years reduce their risk of subsequent weight regain by nearly 50% [7].

Older age at enrollment, lower baseline BMI, and having health insurance that provided a financial incentive for program participation were independently associated with 2-year program retention. Many investigators have reported that older individuals are more likely to adhere to behavioral weight management and intensive lifestyle interventions, perhaps because of fewer competing demands [2,8,9]. Our finding that lower baseline BMI was associated with program retention differs from the findings of other studies [2] and does not appear to be explained by differences in the prevalence of functional limitations or comorbidities. It should be recognized, however, that at baseline, 89% of our study participants were at least moderately obese (BMI 35-39.9 kg/m^2^) and 47% were severely obese (BMI ≥ 40 kg/m^2^). Martin et al have previously demonstrated that retention in a commercial weight loss program was higher when participation was promoted through incentives and when participants enrolled through their work even when they paid out-of-pocket for the program [10]. Our finding that the behavioral economic approach of loss aversion, which takes away an existing benefit when an individual fails to achieve a health goal rather than rewarding an individual for achieving the health goal, improved program uptake and retention and should be tested in other settings [3].

The rate of weight loss over the first 6 months of program participation was greater for 2-year program completers than non-completers, and program completers lost a significantly greater percentage of baseline body weight at both 6 months and 2 years. Indeed, we found that the percent change in body weight as early as 2 weeks after program initiation predicted percent change in body weight at 12 weeks (data not shown). Fabricatore and colleagues and Wadden and colleagues have also reported that early weight loss predicts longer-term weight loss success in the setting of randomized, controlled clinical trials [9,11]. For those who completed our 2 year program, mean weight loss was 15 ± 12 kilograms and mean reduction in BMI was 5.1 ± 4.0 kg/m^2^. There were substantial improvements in cardiovascular risk factors, comorbidities, and health-related quality-of-life among program completers.

Our results differ from those of Tsai and Wadden [1] and Spring and colleagues [2] who reported lesser degrees of initial weight loss, lower patient retention, and greater weight regain among participants enrolled in behavioral weight management programs. The differences in initial weight loss may be explained in part by differences in program structure. Wing et al reported that people who were provided behavioral therapy and an additional modality (e.g. a shopping list or the direct provision of food) did better than those who were provided behavioral therapy alone [7]. Diets that incorporate meal replacements result in predictable weight loss by divorcing patients from unhealthy habits and by making their meals decision-free. Lichtman et al examined reported energy intake, actual energy intake, and total energy expenditure during a 14-day study [12]. Substantial differences were observed between reported and actual energy intake (a mean of >1,000 kcal/day). Failure to lose weight with diet was explained by underestimation and under-reporting of caloric intake and not by low energy expenditure. Indeed, no subject had a total energy expenditure or resting metabolic rate more than 10% below his or her predicted value. Use of meal replacement products provides structure, removes the need to make decisions about food choices, and results in a more predictable number of calories consumed and more predictable reductions in weight.

Although freedom from depression and from depressive symptoms did not predict retention in our clinical program at 2 years, our results related to depression warrant further discussion. In univariate analyses, two-year completers were significantly less likely to report moderate or severe anxiety or depression at baseline and reported fewer depressive symptoms on the IDS-SR than noncompleters. In addition, IDS-SR scores were significantly improved from baseline among participants who completed 2 years in the program in the context of more frequent antidepressant medication use. In a review of psychosocial predictors of weight control, Teixeira and colleagues found that depression, when assessed before treatment, did not predict treatment outcomes [13]. Fabricatore and colleagues have shown that in a clinical trial setting, fewer depressive symptoms at baseline predict weight loss success at one year [9]. Elder and colleagues have demonstrated a strong correlation between weight loss and reduction in depression [14] and Wing and Phelan have demonstrated that lower levels of depression are related to greater odds of weight loss maintenance [7]. These results suggest that identifying and treating depression, especially during and immediately after the transition from VLCD to regular food stuffs, may be an important determinant of weight loss maintenance and program retention.

## Conclusion

In summary, we have demonstrated that a physician-led, multidisciplinary, clinical, weight management program that employs short-term meal replacement for weight loss induction and intensive behavioral therapy and financial incentives for longer-term weight loss maintenance demonstrated both high retention and excellent outcomes. Older participants and those with moderate as compared to severe obesity were more likely to complete the 2-year clinical program. Participants with less anxiety and depression and less severe symptoms of depression also tended to be more likely to complete the 2-year program although the results were not statistically significant in multivariate analysis. Future interventions should further explore the relationship between depression and program retention.
